# Exploring Triacylglycerol Biosynthetic Pathway in Developing Seeds of Chia (*Salvia hispanica* L.): A Transcriptomic Approach

**DOI:** 10.1371/journal.pone.0123580

**Published:** 2015-04-13

**Authors:** Sreedhar R. V., Priya Kumari, Sunny D. Rupwate, Ram Rajasekharan, Malathi Srinivasan

**Affiliations:** 1 CSIR-Lipidomic Centre (CSIR-LIPIC), CSIR-Central Food Technological Research Institute (CSIR-CFTRI) Resource Centre, Allalasandra, GKVK Post, Bangalore—560 065, Karnataka, India; 2 CSIR-Lipidomic Centre (CSIR-LIPIC), CSIR-Central Food Technological Research Institute (CSIR-CFTRI), Mysore—570 020, Karnataka, India; 3 Academy of Scientific and Innovative Research, CSIR, New Delhi—110 025, India; Washington State University, UNITED STATES

## Abstract

Chia (*Salvia hispanica* L.), a member of the mint family (Lamiaceae), is a rediscovered crop with great importance in health and nutrition and is also the highest known terrestrial plant source of heart-healthy omega-3 fatty acid, alpha linolenic acid (ALA). At present, there is no public genomic information or database available for this crop, hindering research on its genetic improvement through genomics-assisted breeding programs. The first comprehensive analysis of the global transcriptome profile of developing *Salvia hispanica* L. seeds, with special reference to lipid biosynthesis is presented in this study. RNA from five different stages of seed development was extracted and sequenced separately using the Illumina GAIIx platform. *De novo* assembly of processed reads in the pooled transcriptome using Trinity yielded 76,014 transcripts. The total transcript length was 66,944,462 bases (66.9 Mb), with an average length of approximately 880 bases. In the molecular functions category of Gene Ontology (GO) terms, ATP binding and nucleotide binding were found to be the most abundant and in the biological processes category, the metabolic process and the regulation of transcription-DNA-dependent and oxidation-reduction process were abundant. From the EuKaryotic Orthologous Groups of proteins (KOG) classification, the major category was “Metabolism” (31.97%), of which the most prominent class was ‘carbohydrate metabolism and transport’ (5.81% of total KOG classifications) followed by ‘secondary metabolite biosynthesis transport and catabolism’ (5.34%) and ‘lipid metabolism’ (4.57%). A majority of the candidate genes involved in lipid biosynthesis and oil accumulation were identified. Furthermore, 5596 simple sequence repeats (SSRs) were identified. The transcriptome data was further validated through confirmative PCR and qRT-PCR for select lipid genes. Our study provides insight into the complex transcriptome and will contribute to further genome-wide research and understanding of chia. The identified novel UniGenes will facilitate gene discovery and creation of genomic resource for this crop.

## Introduction

A low ratio of ω-6/ω-3 fatty acids (5 or lower) in the diet is considered healthy for combating cardiovascular diseases, cancer, inflammation and autoimmune diseases. However, this ratio averages 16.74 in the US, 15 in the UK and northern Europe and, alarmingly, as high as 38–50 in urban India [1]. Flax seed, marine algae and fish are potent sources of ω-3 fatty acids, but their use is limited as a result of certain anti-nutritional factors, fishy flavor, low availability, contamination with mercury compounds and non-vegetarian nature [2]. Chia (*Salvia hispanica* L.), a member of the mint family (Lamiaceae), is native to Mexico and parts of South America. The seeds contain approximately 26–35% oil by weight and have the highest known content of the omega-3 fatty acid, α-linolenic acid (approximately 60% of the total fatty acids) [3]. Chia oil, with very high omega-3 fatty acid (α-linolenic acid/ ALA) content, represents an excellent alternative source of this nutritive compound. Chia oil has a ω-6/ω-3 fatty acid ratio of <1, and regular use can assist in obtaining a healthy overall dietary ratio. α-Linolenic acid is a known precursor for the synthesis of very long-chain polyunsaturated fatty acids such as eicosapentaenoic acid and docosahexaenoic acid, which have an important role in brain development, cognition, behavior and eyesight [4] and reduce the risk of coronary heart diseases [1]. Chia seeds are also a rich source of protein (up to 26%) [3]; total dietary fiber (up to 40%) [5]; antioxidants; essential minerals such as calcium, magnesium, phosphorus, iron, zinc and copper; and vitamins such as thiamine, niacin, riboflavin, vitamin C and vitamin E [6,7], which potentially supports their reputation as a ‘superfood.’

Chia was an important staple food crop of pre-Columbian Central America along with maize, amaranth and beans. This crop was re-discovered recently due to its superior nutritional qualities. Chia, which is considered a dietary nutritional supplement, is safe and is exempted from regulation by the US Food and Drug Administration. The European Commission authorizes the sale of chia seeds in the Union as a novel food ingredient with a daily intake of up to 15 grams, and chia seeds can be used in baked products, breakfast cereals, and fruit, nut and seed mixes in levels of up to 10% [8]. The seeds can be eaten raw as whole or ground seeds or as sprouts. The seed flavor is very mild, and can be considered almost flavorless. The seeds can be added to almost anything, including cereals, salads, soups, yogurt, sweets, baby foods, health bars, breads, cakes and other foods. Commercially available products made with chia include sports nutrition bars, breakfast bars, chocolate bars, oil soft-gel capsules, seed flour and various types of cakes, breads and other bakery products. Presently, chia is cultivated commercially in Argentina, Australia, Bolivia, Ecuador, Guatemala and Mexico [9]. We have for the first time, successfully evaluated its adaptability and suitability for commercial cultivation under tropical South Indian conditions. We are currently in the process of genetically improving this crop to develop lines with a high yield, higher oil content and modified fatty acid profiles to be suitable for cooking and to meet other nutritional requirements.

Modern molecular breeding techniques with biotechnological intervention have revolutionized the process of genetic improvement in plants. Various genomic tools have greatly facilitated the development of improved genotypes or varieties in several crops. Whole-genome crop sequences are very useful in crop improvement programs. With high resource and time demands, the generation of these sequences has been restricted to the most important crops. Transcriptome sequencing by next-generation sequencing (NGS) technologies and analysis with powerful bioinformatic tools has become a fast and inexpensive alternative, especially for non-model or orphan crop species in which reference genomes or sequence data are unavailable. The generation of large-scale expressed sequence tags (ESTs) or transcript data and molecular markers have accelerated breeding research in these crops. The availability of a large number of genetic markers developed through NGS technologies is facilitating trait mapping and linkage mapping, making marker-assisted breeding more feasible [10].

In addition to aiding in genomics-assisted breeding programs, transcriptomic analysis through NGS can facilitate the genetic modification of plants. Genetic engineering of plants to modify their nutritional compositions has been successful, particularly in oil-seed crops such as canola, brown mustard, soybean, safflower and linseed [11]. Transgenic safflower with seed oil containing >70% gamma linolenic acid (GLA) [12] has been recently commercialized in the USA, and transgenic soybeans producing 15–30% stearidonic acid (SDA) and 5–8% GLA developed by Monsanto and Solae LLC (Limited Liability Company) are close to commercialization after achieving successful safety assessments and GRAS (generally recognized as safe) notice [13]. Oil deposition has been also enhanced by the over-expression of diacylglycerol acyltransferase (AtDGAT) cDNA in wild-type *Arabidopsis thaliana* [14] and by the up-regulation of two soybean Dof-type transcription factor (GmDof) genes associated with fatty acid biosynthesis [15].

A prerequisite for introducing successful and stable genetic manipulation is to understand the transcribed gene and pathway information. Insight into the spatial and temporal control of gene expression and an integrated model involving biochemical pathways and differentially expressed genes (DEGs) during lipid biosynthesis can reveal the gene expression profile, important genes and their levels of expression. Such insight helps to identify, characterize and modify the potential transcripts of interest.

With no public genomic information (except for a partial nucleotide sequence of the chloroplast *rbcL* gene for rubisco; GenBank: Z37442.1) or database entries available for chia, our major aim was to establish a public information platform for further functional genomic studies. We also aimed at identifying the transcripts involved in lipid biosynthesis. In the present study, we present the first comprehensive analysis of a global transcriptome profile of developing chia seeds. We used Illumina GAIIx sequencing platform to sequence the transcriptome and to investigate gene expression in developing seeds of chia. To provide the most comprehensive representation of the developing seed transcriptome, we extracted and separately sequenced RNA from five different stages of seed development (3, 7, 14, 21 and 28 days after flower opening), assuming differential gene expression levels through the developmental stages of the seeds.

## Results and Discussion

With the aim of characterizing the transcriptome of developing seeds of *S*. *hispanica*, we sequenced five cDNA samples from five different stages of seed development using the Illumina GAIIx platform. Each run yielded approximately 35 million raw reads, ultimately yielding 3.5GB data. The raw paired-end sequence data in the FASTQ format was deposited in the National Center for Biotechnology Information’s (NCBI) BioProject database (as a Short Read Archive; SRA) under accession number PRJNA196477. A pooled data set was created by combining the reads from five different sequenced libraries and subjected to *de novo* assembly. *De novo* assembly of processed reads using Trinity yielded 76,014 transcripts after filtering out those shorter than 200 bases. The details of the pooled transcriptome are provided in Table 1.

**Table 1:** Transcriptome summary statistics.

Total number of transcripts	76,014
Minimum transcript length (bp)	201
Maximum transcript length (bp)	11,579
Average transcript length (bp)	880.69
Total transcript length (bp)	66,944,462
Transcripts >500 bp	43,435
Transcripts >1 kbp	24,754
Transcripts >5 kbp	129
N50 contig size (bp)	1338
GC %	45.81

The total transcript length was 66,944,462 bases (66.9 Mb), with an average length of approximately 880 bases. The transcripts were 54.19% AT, which was marginally higher than the GC content of 45.81%. There were 24,754 transcripts with a length of more than 1000 bp. In another species of Salvia (*Salvia splendens*) [16], *de novo* assembly and annotation of transcriptome yielded 83,093 and 81,127 transcripts in two different strains (strain 35 and Cailinghong) with average length of 905 and 919 bases, respectively. A total of 28,197 and 27,992 transcripts, respectively, were more than 1000 bp. There was also difference in the GC content which was approximately 50% in *S*. *splendens* transcriptome compared to 45.81% in *S*. *hispanica*. The N50 value of our assembly (1338 bases) was almost similar to that of *S*. *splendens* (1346 bases) [16]. However, N50 contig size of chia assembly was 1338 bases which is higher than most of the recently published plant transcriptome assemblies like celery (1204 and 1193 bases) [17], *Houttuynia cordata* Thumb. (1051 bases) [18], Chinese hazelnut (799 bases) [19] and Geraniaceae (478 and 1319 bases) [20], indicating good transcriptome assembly. In medicinal plant *Salvia miltiorrhiza*, the transcriptome sequencing of leaf and root tissues using 454 GS-FLX system and its assembly yielded 64,139 unigenes with average length of 413 bases [21]. The N50 value of isotigs was 750 bases. Sequencing of transcriptome of *Salvia sclarea* L calyx using 454 GS-FLX system resulted in identification of 45,822 unique sequences with average read size of 214 bp [22]. The average GC content was 45% which is similar to that of our present study in *S*. *hispanica*.

### Functional annotation and candidate genes encoding enzymes involved in lipid biosynthesis

Functional annotation of the pooled transcripts was conducted using BLASTX (E value≤0.001) against the UniProt Plant Protein database, which resulted in 83% of the transcripts being annotated. A total of 27,122 unique genes were mapped. The top five homologous plant genomes contributing to predicted transcript annotation were *Vitis vinifera* (44%), *Populus balsamifera* (22%), *Ricinus communis* (19%), *A*. *thaliana* (3%) and *Glycine max* (3%) (S1 Table). A similar trend was also observed in *S*. *splendens* [16]. The top proteins contributing were pentatricopeptide repeat-containing protein-putative (18%), DNA binding protein-putative (13%), an uncharacterized protein (12%), protein-binding protein-putative (11%), transcription factor-putative (9%), zinc finger protein-putative (8%) and Cytochrome P450 (8%). There were additional homology searches made using BLASTX to further annotate genes in transcriptome by using known sequence of lipid related genes like peanut soluble MGAT [23].

**Fig 1:**
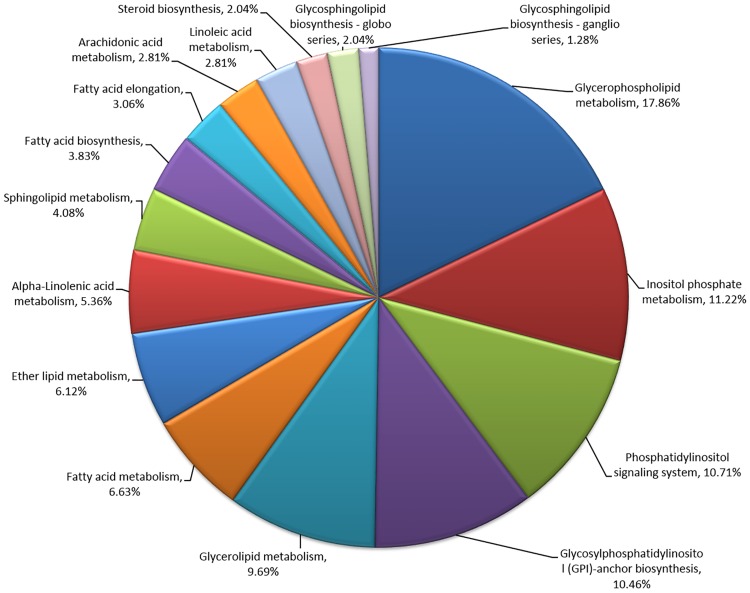
Percentage distribution of lipid synthesis-related transcripts.

Pathways contributing to lipid biosynthesis and modification have been well documented in major oil seed crops. Through our study, most of the known enzymes involved in lipid biosynthesis were successfully identified in chia based on the annotation of the transcripts obtained. The percentage distribution of lipid biosynthesis-related transcripts identified in chia is provided in Fig 1. Glycerophospholipid metabolism, inositol phosphate metabolism, the phosphatidylinositol signaling system, glycosylphosphatidylinositol-anchor biosynthesis and glycerolipid metabolism-related transcripts were the highest in number, and transcripts related to α-linolenic acid metabolism, linoleic acid metabolism and arachidonic acid metabolism were also prominent.

### Metabolic pathway related to oil accumulation

Triacylglycerol (TAG) is biosynthesized *de novo* by a series of enzymatic acylation reactions of the glycolytic intermediate glycerol-3-phosphate (G3P) that are acyl-CoA dependent. Certain dephosphorylation steps are also required for TAG synthesis.

**Fig 2:**
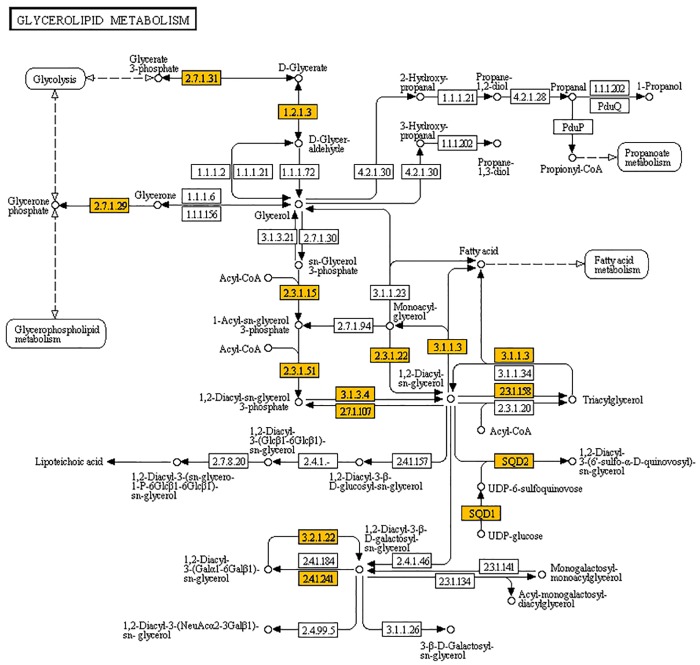
Glycerolipid metabolism pathway. Genes highlighted are those identified in chia in the present study.

Coding DNA sequences (CDS) encoding enzymes in the TAG biosynthetic pathway were identified in the transcriptome, including those in both the Kennedy pathway [24, 25] and the soluble TAG biosynthesis pathway [23] (Fig 2). The biosynthetic machinery responsible for the formation of TAG must compete with phospholipid biosynthesis for a common intermediate precursor, i.e., phosphatidic acid (PA). In yeast, carbon flux in these pathways was recently shown to be regulated by a phospholipase C [26]. The exploration of such regulation in plant systems like chia, using its transcriptome data, could be interesting. The first step in the biosynthesis of glycerolipid/glycerophospholipid is the acylation of glycerol-3-phosphate (G3P) at its two free hydroxyl positions to produce lysophosphatidic acid (LPA) and then PA by the enzymes G3P acyltransferase (GPAT) and LPA acyltransferase (LPAT), respectively. Both of these products can be dephosphorylated to monoacylglycerol (MAG) and diacylglycerol (DAG); PA is dephosphorylated by phosphatidic acid phosphatase (PAP). DAG is then acylated to produce TAG. The PA-independent pathway leading to the formation of TAG from DAG via MAG involves two acylation steps after the action of lipid phosphatase on LPA [27]. The acylation of monoacylglycerol by MAG acyltransferase (MGAT) has been described in plants, and it is interesting to note that the acylation is carried out by two types of enzymes, a soluble MGAT [27] and the oil body-associated protein, oleosin. Previously, oleosins were thought to be structural proteins, but recently, they have been shown to exhibit MGAT and phospholipase activities [28]. In our analysis of the chia transcriptome, four CDS corresponding to oleosin and two CDS corresponding to soluble MGAT were identified.

In oilseeds like chia, TAG is sequestered in the oil bodies, which are made up of a hydrophobic core mainly comprising TAG and sterol ester that is surrounded by a phospholipid monolayer containing proteins such as oleosin, steroleosin and caleosin, which are vital for the appropriate accumulation and storage of TAG. Many other proteins play crucial roles in supporting TAG biosynthesis, including the oil body-associated protein discussed above, acyl carrier protein and other seed-specific proteins.

The mono-unsaturated fatty acids (MUFAs) are synthesized in plastids, and their further elongation and desaturation occur in the endoplasmic reticulum. MUFAs present in phosphatidylcholine (PC) at the *sn*-2 position are converted to poly-unsaturated fatty acids (PUFAs) by certain desaturases. These PUFAs are channeled into TAG through a unique step in which the PUFAs from the PC molecule are transferred to DAG to produce TAG by phospholipid:diacylglycerol acyltransferase (PDAT). This reaction is unique because it utilizes PC as the acyl donor, whereas in other acylation steps, acyl-CoA acts as the acyl donor. In addition to TAG, the other product of the PDAT reaction is lyso-phosphatidylcholine (LPC), which is converted to PC by acyl-CoA:lyso-phosphatidylcholine acyltransferase (LPCAT). This reaction must occur for PDAT to function [29, 30]. PC is not only a membrane lipid but also plays an important role in the production of PUFAs by acting as a substrate for various desaturase-type enzymes. PC becomes even more relevant when studying the enzymes related to PC acyl chain editing in chia because chia accumulates high amounts of ALA. CDS corresponding to PDAT were detected in the chia transcriptome, and this enzyme might play a role in incorporating specific fatty acids into the TAG pool.

**Fig 3:**
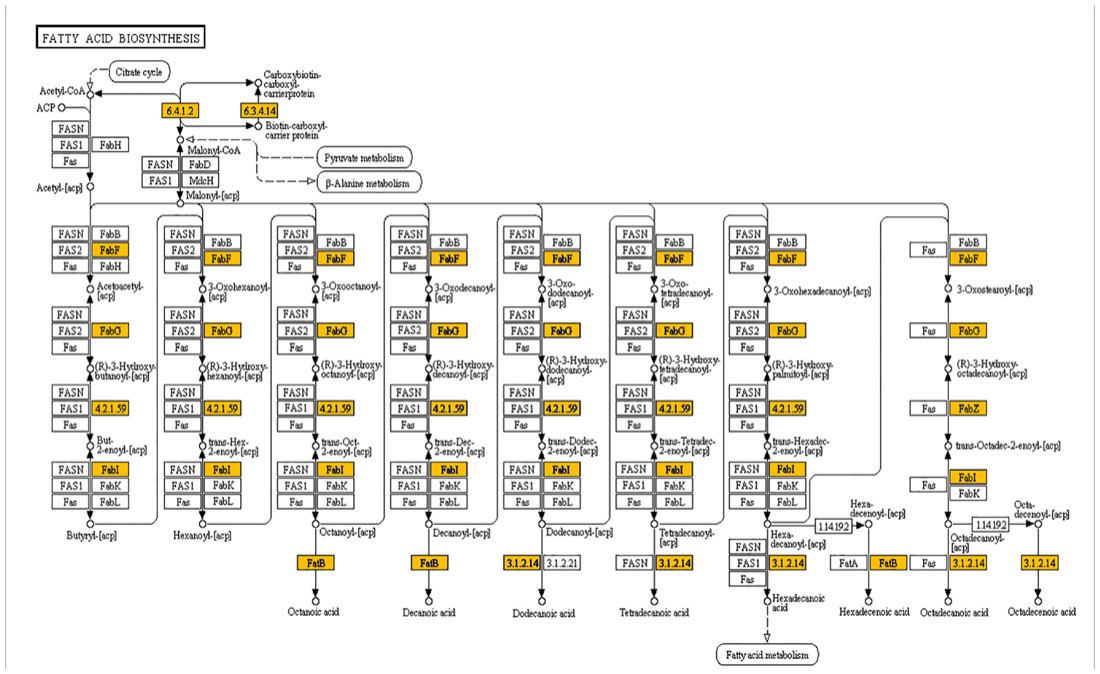
Fatty acid biosynthesis pathway. Genes highlighted are those identified in chia in the present study.

CDS encoding enzymes for the fatty acid biosynthetic pathways (Fig 3) were identified in the *S*. *hispanica* transcriptome. The first step for fatty acid biosynthesis involves acetyl-CoA carboxylase, which catalyzes an interesting reaction in which CO_2_ is transferred from bicarbonate to acetyl-CoA to produce malonyl-CoA. This reaction is followed by the transfer of a malonyl group to ACP by malonyl-CoA:ACP transacylase. This malonyl group is subsequently elongated by a sequence of condensation reactions with either acyl-ACP or acetyl-CoA by multiple isoforms of β-ketoacyl-ACP synthase. Furthermore, CDS encoding fatty acid desaturase enzymes were also identified. The other family of enzymes that can dictate the fatty acid profile of an organism is acyl-ACP thioesterases, and the corresponding reads were identified in the chia transcriptome.

### Gene Ontology (GO) annotation

**Fig 4:**
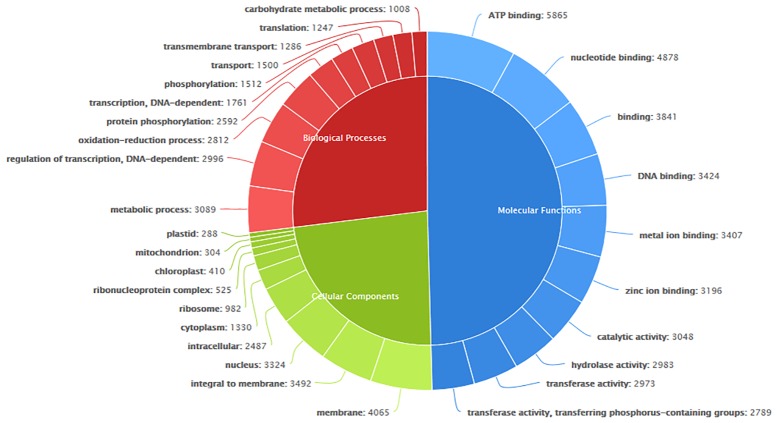
GO classification. The top 10 GO terms in Cellular components, Molecular function and Biological processes are displayed.

GO is used widely for the standard representation of genes across species and to provide a controlled vocabulary of terms for describing gene products [31]. The contigs from the pooled transcriptome were assigned GO terms based on BLASTP searches against sequences with previously identified products (S2 Table). The three categories of GO terms (biological processes, cellular components and molecular functions) were represented by 870, 236 and 1203 terms, respectively (Fig 4). A similar pattern was observed in *S*. *miltiorrhiza* transcriptome [21]. The top ten GO terms in each component are also shown. In the molecular functions category, ATP binding and nucleotide binding were found to be the most abundant terms, whereas in the biological processes category, the metabolic process and the regulation of transcription-DNA-dependent and oxidation-reduction process were abundant. Predominance of metabolic process terms indicates metabolism as major process in developing seeds. In *S*. *splendens*, biological process ontology distribution pattern indicated multiple developmental processes [16] which may be due to the transcriptome analysis of tissues from leaf, stem, shoot and root tissues. Membrane, integral to membrane and nucleus classes were prominent GO terms in the cellular components category.

### KOG annotation

**Fig 5:**
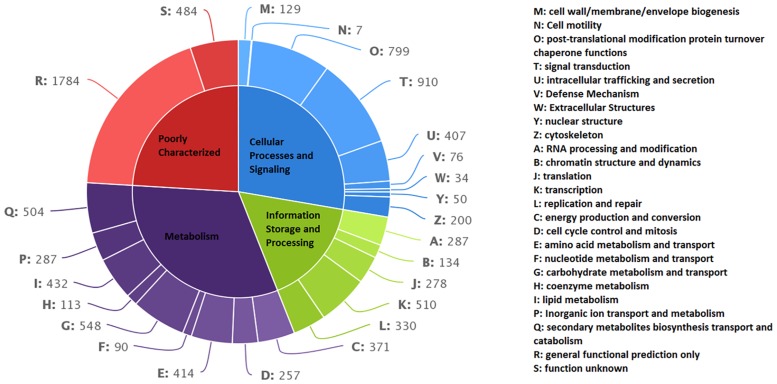
KOG classification. KOG functional categories of the transcripts after annotation against the KOG proteins.

KOG terms derived from the annotation of 8,288 transcripts using KOG proteins from eukaryotic clusters are provided in S3 Table. The KOG classifications with multiple assignments were individually assessed and assigned to transcripts (individual KOG assignment; the redundant transcript list is shown in Fig 5). From the KOG classification, the major category was “Metabolism” (31.97%), of which the most prominent class was ‘carbohydrate metabolism and transport’ (5.81% of total KOG classifications) followed by ‘secondary metabolite biosynthesis transport and catabolism’ (5.34%) and ‘lipid metabolism’ (4.57%). It is noteworthy that in the “metabolism” category, the ‘carbohydrate metabolism and transport’ and ‘lipid metabolism’ classes were the best represented, highlighting the data integrity at the sequencing and analysis levels. Developing chia seeds accumulate carbohydrates and lipids at high levels. This phenomenon is reflected in proteins related to metabolism being prominently represented in the KOG classification. The second major category was “Cellular processes and Signaling” (27.68%), in which the most frequently observed classes were ‘signal transduction’ (9.64%), ‘post-translational modification protein turnover’ (8.47%) and ‘intracellular trafficking and secretion’ (4.31%). In the “Information storage and processing category”, the highly occurring classes were ‘transcription’ (5.41%), ‘replication and repair’ (3.5%) and ‘RNA processing and modification’ (3.04%). Of the “Poorly characterized” category annotations, ‘general functional prediction only’ represented 18.91% and ‘function unknown’ represented 5.1% of the transcripts. The prominent presence of the “Poorly characterized” category may have occurred because chia is distantly related to the organisms comprising the eukaryotic KOG database.

### Validation of Transcriptome

**Fig 6:**
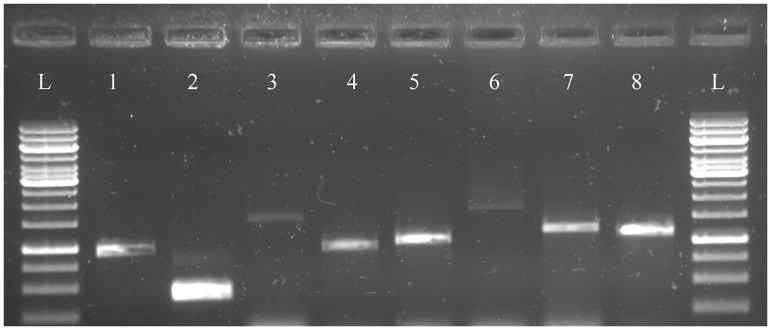
Detection and validation of different lipid genes by PCR. cDNA was used as template to amplify lipid genes. The amplified fragments were analyzed by 0.8% agarose gel electrophoresis; 1 kb DNA ladder containing 14 bands (from down 250, 500, 750, 1000, 1500, 2000, 2500, 3000, 3500, 4000, 5000, 6000, 8000, 10000 bp; GeneRuler 1 Kb DNA ladder from Thermo Scientific, cat no. SM0311) was used. Lane L: 1 kb DNA ladder, Lane1: *MGAT* (963 bp), Lane2: *OLE1*(429 bp), Lane3: *DGAT1*(1620 bp), Lane4: *DGAT2* (1014 bp), Lane5: *DGAT3* (1098 bp), Lane6: *PDAT* (1881 bp), Lane7: *Desaturase* (1300 bp), Lane8: *Thiolase* (1179 bp)

Since transcript assembly needs validation and as our interest is in chia lipid metabolism, we chose to validate eight lipid genes. The transcripts of all the 8 lipid genes, viz., *MGAT*, *OLE1*, *DGAT1*, *DGAT2*, *DGAT3*, *PDAT*, *Thiolase* and *Desaturase* were confirmed by PCR, as was observed in an agarose gel. The amplicon sizes matched with the expected size of the gene based on assembled transcripts (Fig 6). *MGAT*, *OLE1*, *DGAT2*, *DGAT3*, *Delta-15-desaturase and omega-3 desaturase* PCR products were further sequenced for confirmation, giving ~100% identity with the assembled transcriptome sequences (S1 File). This is the first time that these genes have been identified in *S*. *hispanica*. The sequences have been deposited at NCBI as mRNA sequences with the accession numbers, GenBank: KM016233 and GenBank: KM016234 for *OLE1* and *MGAT*, respectively. Further, the protein sequences of *MGAT*, *OLE1*, *DGAT2*, *DGAT3*, *Delta-15-desaturase and omega-3 desaturase* of chia were aligned with known sequences of other oilseed plants for homology (S2 File).

### Determination of mRNA Copy Number

**Fig 7:**
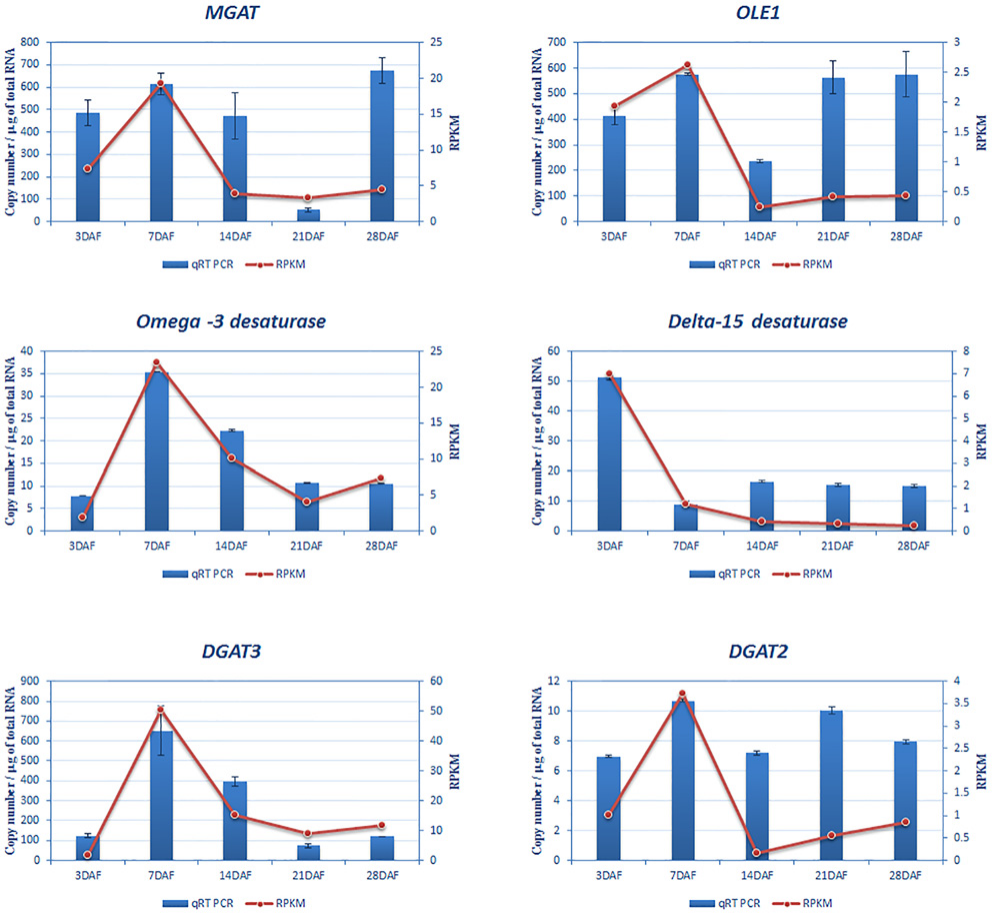
Real Time PCR analysis to validate the transcriptome data. Validation of expression pattern of transcriptome of *OLE1*, *MGAT*, *DGAT2*, *DGAT3*, *Delta 15 desaturase* and *Omega-3 desaturase* by quantitative qRT-PCR was carried out using total RNA isolated from different stages of Chia seeds (3, 7, 14, 21 and 28 DAF). Absolute copy number of transcripts was calculated. Error bars represent standard deviation between three replicates. Expression value RPKM obtained by NGS for same stages is also represented.

Quantitative Reverse transcriptase polymerase chain reaction based method was used for absolute quantification of copy number of mRNA of the genes *MGAT*, *OLE1*, *DGAT2*, *DGAT3*, *Delta-15-desaturase and omega-3 desaturase*. A standard curve constructed by using linear DNA as template, was highly linear for all genes (R^2^ value was ≥0.98). PCR amplification efficiency (E) was more than 85% and the slopes of the standard curves were between 3.2 and 3.7. The expression levels of the six genes across the various developmental stages as determined by qRT-PCR were generally in agreement with the levels observed using NGS sequencing (Fig 7, S4 Table).

### SSR mining

**Fig 8:**
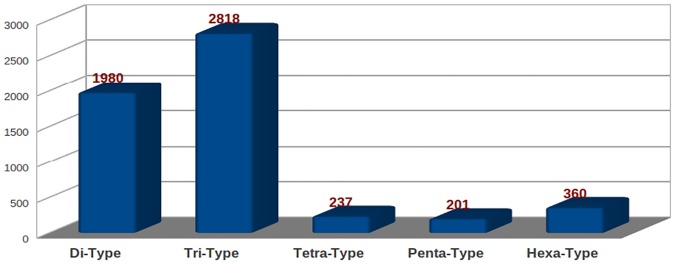
Number of each type of SSR detected in chia transcriptome.

**Fig 9:**
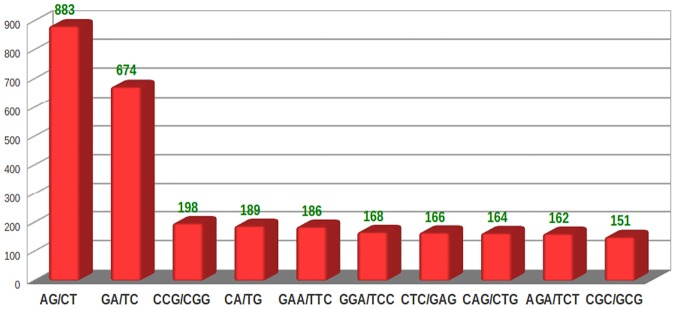
Frequency of classified repeat types.

Microsatellite or SSR markers are the repeats of 2–6 nucleotide bases. These markers are the most important molecular markers, with broad applications in many genetic and genomic studies. The development of EST-SSR markers through transcriptome sequencing has greater advantages than genomic SSRs due to their direct association with transcribed genes and inexpensive development. EST-SSRs are more likely to be tightly linked to a specific gene function and may play direct roles in controlling important agronomic characteristics [32]. EST-SSRs are expected to be more transferable than random genomic SSRs across lines, populations and species [33]. In the present study, 5596 SSRs were identified in 5415 transcripts (S5 Table), out of which 454 sequences contained more than one SSR. The most abundant SSRs were trinucleotide type motifs (2818, 50.4%) followed by dinucleotide type motifs (1980, 35.4%), hexanucleotide type motifs (360, 6.4%), tetranucloetide type motifs (237, 4.2%) and pentanucleotide type motifs (201, 3.6%) (Fig 8). On the contrary, out of the 2,453 SSRs discovered, dinucleotide repeat motifs (39.9%) were the most abundant followed by trinucleotide (29.3%), tetranucleotide (0.8%), pentanucleotide (0.7%) and hexanucleotide (0.7%) repeat motifs in *S*. *splendens* [16]. This may be mainly because of species variation or difference in the tissue used for transcriptome sequencing. The most prominent repeat motif type was (AG/CT) followed by (GA/TC), (CCG/CGG), (CA/TG), (GAA/TTC), (GGA/TCC), (CTC/GAG), (CAG/CTG), (AGA/TCT) and (CGC/GCG) (Fig 9). Chia, being an oil seed plant, we also identified the transcripts related to TAG biosynthesis that have SSRs, most of which were highly expressed throughout the various developmental stages (S6 Table). In a non-model plant such as *S*. *hispanica* that has no genomic sequences available; SSRs such as those mined in our study can be invaluable resource for genetic improvement and the construction of genetic map.

### Developmental stage based differences in gene expression levels

Although we have provided a comprehensive account of the chia transcriptome pooled from 5 different seed developmental stages, detailed analyses of the assembled contigs in each developmental stage is underway. For instance, we have analyzed the differential gene expression levels, especially of genes involved in lipid metabolism, across the five stages. Heat maps representing the gene expression levels of the TAG biosynthetic genes like LPAT, GPAT, MGAT, DGAT etc. and of the fatty acid biosynthetic genes like desaturases and elongases across the five stages of chia seed development have been provided (S7 and S8 Tables).

## Conclusions

For the first time, we have established a public genomic information platform for chia (*S*. *hispanica* L.), a rediscovered crop of immense health and nutritional importance that is also the best known plant source of omega-3 fatty acids. This comprehensive transcriptome analysis data can form the basis for accelerated genomics-assisted genetic improvement programs and facilitate a better understanding and more effective manipulation of biochemical pathways, including lipid biosynthesis. The data across five different seed developmental stages, with special reference to genes involved in lipid metabolism can serve as very valuable information, especially in plant lipid research focusing on improving the quality and quantity of chia seed oil and in promoting it as commercial edible oil. This knowledge can subsequently enable more focused and intensive research on this underutilized crop, leading to the development of better cultivars or lines promoting its large-scale commercial cultivation and utilization, especially in developing countries with rampant malnutrition.

## Materials and Methods

### Ethics statement

No specific permissions were required for these locations or activities and the study did not involve any endangered or protected species, since plants were grown in our own private field at CSIR-Central Food Technological Research Institute Resource Centre (CSIR-Central Institute of Medicinal and Aromatic Plants Research Farm) in Bangalore located at 12°58' N latitude and 77°35' E longitude with an altitude of 930 msl and annual mean rainfall of 870 mm in red loamy soil. As a result of our genetic improvement program involving chia, a promising, high-yielding line producing blue flowers and white seeds (CHIAmpion B1) has been developed by us and was used in this study. It is not an endangered species.

### Total RNA extraction, library construction and deep sequencing

Developing seeds at 3, 7, 14, 21 and 28 days after flower opening (DAF) were collected from five different plants and the samples were pooled. The 28 days after flower opening seeds represent completely matured embryo or seed. RNA from each pool was isolated using a Qiagen RNeasy Mini Kit following the manufacturer’s protocol. The yield and quality were determined using an Agilent 2100 Bioanalyzer and a NanoDrop spectrophotometer. For RNA library construction and deep sequencing, equal quantity of RNA from each pool was used. Approximately 6 μg of RNA representing each group was sequenced using the Illumina GAIIx platform. The workflow of the present study is represented in S1 Fig.

### Raw data preprocessing

Raw reads were subjected to quality check using SeqQC. High-quality bases (Q≥20) comprised more than 85% of the bases of both the forward and reverse paired-end reads. The raw data was filtered to remove adaptor reads and low-quality (Phred Score<20) reads using the NGS QC Toolkit [34], yielding a dataset consisting of clean reads.

### *De novo* transcriptome assembly

High-quality, filtered paired and orphan sequence reads (Phred Score≥20) obtained from all 5 samples were pooled to serve as a representative transcriptome and were provided as an input for transcriptome assembly. Trinity Assembler [35] was used to generate unique transcripts (UTs). Trinity assembly is based on the de Bruijn graph [36]. The assembled UTs with sequence lengths longer than 200 bp were considered for downstream transcript annotation and quantification.

### Annotation and quantification of the transcriptome

Annotation for all the UTs (≥200 bp) was performed using a BLAST [37] homology search against the UniProt Plant Protein Database [38]. Blast hits with an E-value of ≤1e-3 were considered annotated homologous proteins. GO and KEGG pathway information for the annotated transcripts was assigned based on the homologous proteins. The expression levels of all the unique transcripts (UTs) in individual libraries (3, 7, 14, 21 and 28 DAF) were measured by mapping the HQ filtered reads using the BOWTIE2 tool [39] with final assembled UTs as reference and calculating the RPKM (reads per kilobase per million reads) [40] values based on the mapped reads and the coverage of individual UTs. The RPKM method corrects for biases in total gene exon size and normalizes to the total number of short-read sequences obtained in each library. The fold expression of the UTs was further obtained by calculating the Log base 2 of RPKM values.

### Validation of Transcriptome

To assess the precision and quality of the assembly and annotation data that were derived from the Illumina sequencing, PCR amplification with gene specific primers for eight lipid genes (*MGAT*, *OLE1*, *DGAT1*, *DGAT2*, *DGAT3*, *PDAT*, *Thiolase* and *Desaturase*) that are known to be involved in plant lipid metabolism, using cDNA as template was carried out. Total RNA was extracted from immature chia seeds using TRI Reagent (Sigma). The cDNA was synthesized from 1μg of total RNA using SuperScript III First-Strand Synthesis System (Invitrogen) with oligo dT primers. A set of gene specific primers for the eight genes to be assessed were designed based on the transcriptome sequence and were used in a polymerase chain reaction with the above cDNA as the template, in order to validate the assembled sequence. The primer sequences are provided in Table 2. PCR condition used is as follows: 95°C for 1 min. followed by 35 cycles of 95°C for 30 sec, annealing temperature for 30 sec, extension temperature 72°C for 2 min., followed by final extension 72°C for 5 min. The PCR products were electrophoresed using a 0.8% agarose gel. Further, the PCR amplicons of *MGAT*, *OLE1*, *DGAT2*, *DGAT3*, *Delta-15-desaturase and omega-3 desaturase* were Sanger sequenced and compared with the assembled sequences to confirm their identities.

**Table 2:** List of PCR primers used for validation.

Gene name	Primer sequence	Expected size (bp)	Location of amplicon	T_a_ (°C)
***MGAT***	FW: 5’ ATGTCGCCGGAAAATCC 3’	963	Full length	58
	RV: 5’ ATTCTTCTTACCATATCTCTCAACT 3’			
***OLE1***	FW: 5’ ATGGCTGATCAACACTACGG 3’	429	Full length	58
	RV: 5’ AGAGCTTTGCGCAACGG 3’			
***DGAT1***	FW: 5’ ATGGCGATCACGGACTC 3’	1620	Full length	62
	RV: 5’ CCTTGTGCCAGCTTTTCG 3’			
***DGAT2***	FW: 5’ATGTCGTCTGAATCCAACGG 3’	1014	Full length	50
	RV: 5’ TAGAATCCTAAGCTGCAAGTCTG 3’			
***DGAT3***	FW: 5’ ATGGACGCCGCTGCCATG 3’	1098	Full length	65
	RV: 5’ AGATGCAGCAGCAAAACCAA 3’			
***PDAT***	FW: 5’ AAGAAGCAGCGGAAATGGT 3’	1881	91–1871	55.9
	RV: 5’ GATCCTTTCGGACCACTTGA 3’			
***Thiolase***	FW: 5’ TGGAAAAACACCAGGAAAGG 3’	1179	63–1241	50
	RV: 5’ CCAAAGCTTCCGATCTTGTC 3’			
***Desaturase***	FW: 5’ TAGACTCGCACACTCTGGCTTT 3’	1300	9–1308	65
	RV: 5’ AAGGAATGTGATGGGCTGAG 3’			

### Real time Quantitative Reverse Transcriptase PCR

We confirmed the mRNA expression levels of six lipid genes, viz., *MGAT*, *OLE*, *DGAT2*, *DGAT3*, *Delta-15-desaturase and* ω*-3 desaturase* in seeds collected from five different stages by qRT-PCR. The mRNA samples were prepared from seeds and were used to synthesize the cDNA. 5μg total RNA was used with Super Script First-Strand (Invitrogen; cat.no. #11904–018) with oligo dT primer. A set of gene specific RT primers for all six genes were designed to quantify the copy number. 20μl qRT-PCR reaction mixture containing 1μl of template cDNA, 0.8μl (200nM) each of forward and reverse primers and 10μl of SsoFast Evagreen Super mix (Bio-Rad; cat.no#172-5201AP) was used. The primers used for qRT-PCR are shown in Table 3 with their respective Ta and amplicon sizes. The Tm was determined by running a gradient PCR from 55–65°C. The reaction was carried out under following conditions: 95°C for 30 sec followed by 40 cycles of 95°C for 1 sec, respective annealing temperature for 5 sec + plate read, go to 2 for 40 times, melt curve, 65°C—95°C (in 0.5°C increments), plate read. The intensity of fluorescence was captured at each cycle using a Real Time System Machine (Bio-Rad, CFX96). A threshold for the increase in fluorescence was arbitrarily set in the exponential phase of the amplification plots, and a standard curve was drawn to show the copy number of the standard DNA (linear) *vs* the threshold cycle.

**Table 3:** Details of qRT PCR primers.

Gene name	Primer sequence for qRT PCR	Ta (°C)	Amplicon length (bp)	Location of amplicon (bp)
*MGAT*	FW: 5’CAAGACGCTGAAGCTGTACG3’	58.3	100	828–927
	RW: 5’AATCCAAGCCCTCATGTCAG3’			
*OLE1*	FW: 5’AGTACATGACCGGGAAGCAC3’	59.4	98	302–399
	RV: 5’GTCCACCCTGTCCTTCATGT3’			
*DGAT2*	FW: 5’ATGCCCGGTTTGTGGAAG3’	61	83	907–989
	RV: 5’AATCCTAAGCTGCAAGTCTGG3’			
*DGAT3*	FW: 5’GCACTTCAAGCAGAGTGCAG3’	61	83	779–861
	RV: 5’CTTGCCTCCCATACACACCT3’			
*Delta 15 desaturase*	FW: 5’CATTTAGTGGAGGCGACGAG3’	50	82	955–1036
	RV: 5’ACGGAACGGCTCCAGATT3’			
ω- 3 *desaturase*	FW: 5’CATGACTGTGGCCATGGAAG3’	50	97	91–187
	RV: 5’ATCCATGGTAGGGCACCAAA3’			

### SSR identification

SSRs were identified using MISA (Micro Satellite identification tool: http://pgrc.ipk-gatersleben.de/misa/), wherein dinucleotide and trinucleotide repeats were given a minimum threshold of 6 and 4 repeats, respectively. A minimum threshold of 3 repeats was given for pentanucleotide and hexanucleotide repeats. The maximum distance between two SSRs was specified as 100 bases.

## Supporting Information

S1 Fig Workflow. (TIF)

S1 File Sequence alignment between transcriptome assembly and Sanger sequencing. (DOCX)

S2 File Sequence alignment of some Chia lipid genes with known oil seed plants. (DOCX)

S1 Table Summary of annotated transcripts of unique genes mapped. (XLSX)

S2 Table Details of Gene Ontology assigned. (XLSX)

S3 Table Summary of KOG annotated transcripts. (XLSX)

S4 Table Expression of all transcripts over different stages of seed development. (XLSX)

S5 Table Details of SSR sequences identified. (XLSX)

S6 Table Transcripts related to TAG biosynthesis having SSRs. (XLSX)

S7 Table Expression levels of some of the genes involved in TAG biosynthesis across the 5 developmental stages. (XLSX)

S8 Table Expression levels of some important genes involved in fatty acid synthesis across the 5 developmental stages. (XLSX)
