# Supplementary material for: Exploring Triacylglycerol Biosynthetic Pathway in Developing Seeds of Chia (Salvia hispanica L.): A Transcriptomic Approach
Source: PLoS One. 2015 Apr 13;10(4):e0123580. doi: 10.1371/journal.pone.0123580 (PMC4395390; doi:10.1371/journal.pone.0123580)
Supplement: S1 File — (DOCX) [file pone.0123580.s002.docx]

**Sequence alignment to show conformity between transcriptome assembly (trans) and Sanger sequencing (seq) of the *in vitro* amplified PCR product**

1. **MGAT**

MGATseq ------------------------------------------------------------

MGATtrans ATGTCGCCGGAAAATCCCTCCAATTTCTGGGGCGATACGCCCGAGGAGGAGTACTACGCC 60

MGATseq ------------------------------------------------------------

MGATtrans TCCCAGGGCGTCCGCAATTCCAAATCCTACTTCGATTCCCCACACGGCCGCCTCTTCACC 120

MGATseq ------TTCCTCCCCCTCGACCCGACCCGCCCCGTCAAGGCCTCCGTCTTCATGACCCAC 54

MGATtrans CAGTCCTTCCTCCCCCTCGACCCGACCCGCCCCGTCAAGGCCTCCGTCTTCATGACCCAC 180

******************************************************

MGATseq GGCTACGGCTCCGACTCCTCCTGGATGTTCCAGAAGTTCTGCATCAGCTACGCCGCCTGG 114

MGATtrans GGCTACGGCTCCGACTCCTCCTGGATGTTCCAGAAGTTCTGCATCAGCTACGCCGCCTGG 240

************************************************************

MGATseq GGCTACGCCGTCTTCGCCGCCGACATGCTCGGCCACGGCCGCTCCGACGGGATCCGCTGC 174

MGATtrans GGCTACGCCGTCTTCGCCGCCGACATGCTCGGCCACGGCCGCTCCGACGGGATCCGCTGC 300

************************************************************

MGATseq TACATGGGCGATCTGCCCAAGGTCGCCGCCGCCTCGCTCGCCTTCTTCAGGAGCGTCAGG 234

MGATtrans TACATGGGCGATCTGCCCAAGGTCGCCGCCGCCTCGCTCGCCTTCTTCAGGAGCGTCAGG 360

************************************************************

MGATseq GTTAGCGACGAGTACAAGGATCTGCCGGCGTTCCTGGTCGGGGAGTCGATGGGAGGGCTC 294

MGATtrans GTTAGCGACGAGTACAAGGATCTGCCGGCGTTCCTGGTCGGGGAGTCGATGGGAGGGCTC 420

************************************************************

MGATseq GCCACGCTGCTCATGTATTTCCAGTCGGAGAAGGATCTGTGGACTGGACTCATCTTCTCC 354

MGATtrans GCCACGCTGCTCATGTATTTCCAGTCGGAGAAGGATCTGTGGACTGGACTCATCTTCTCC 480

************************************************************

MGATseq GCGCCGCTCTTCGTCATTCCCGAGAGCATGATGCCGTCCAAGGTTCACTTGTTCGCGTAC 414

MGATtrans GCGCCGCTCTTCGTCATTCCCGAGAGCATGATGCCGTCCAAGGTTCACTTGTTCGCGTAC 540

************************************************************

MGATseq GGGATGCTATTCGGGTTAGCTGACACATGGGCTGCAATGCCGGACAACAAGATGGTCGGC 474

MGATtrans GGGATGCTATTCGGGTTAGCTGACACATGGGCTGCAATGCCGGACAACAAGATGGTCGGC 600

************************************************************

MGATseq AAGGCGATCAAAGACCCGGAGAAGCTGAAGGTGATCGCGAGCAACCCGATGAGGTACACT 534

MGATtrans AAGGCGATCAAAGACCCGGAGAAGCTGAAGGTGATCGCGAGCAACCCGATGAGGTACACT 660

************************************************************

MGATseq GGGAAACCTAGGGTGGGGACGATGAGGGAGTTGCTGAGGCAGACTGAGTATGCGCAGAAC 594

MGATtrans GGGAAACCTAGGGTGGGGACGATGAGGGAGTTGCTGAGGCAGACTGAGTATGCGCAGAAC 720

************************************************************

MGATseq AACTTCGACAAGGTGACCATCCCCTTCTTCACTGCGCACGGGACATCGGATGGGCTAGCT 654

MGATtrans AACTTCGACAAGGTGACCATCCCCTTCTTCACTGCGCACGGGACATCGGATGGGCTAGCT 780

************************************************************

MGATseq GAGTGGTCCGGATCACAGATGTTGTACGACAAGGCGAGCAGTGAGG-------------- 700

MGATtrans GAGTGGTCCGGATCACAGATGTTGTACGACAAGGCGAGCAGTGAGGACAAGACGCTGAAG 840

**********************************************

MGATseq ------------------------------------------------------------

MGATtrans CTGTACGAAGGGATGTACCACTCTCTGATACAGGGCGAGCCTGACGAGAATGCGAATCTC 900

MGATseq ------------------------------------------------------------

MGATtrans GTGCTGGCTGACATGAGGGCTTGGATTGATGAGAGAGTTGAGAGATATGGTAAGAAGAAT 960

MGATseq ---

MGATtrans TGA 963

1. **OLE1**

OLE1seq ATGGCTGATCAACACTACGGTCAATTCCAATCCAGGCCCCACCATCTCCAGCAGCACCAC 60

OLE1trans ATGGCTGATCAACACTACGGTCAATTCCAATCCAGGCCCCACCATCTCCAGCAGCACCAC 60

************************************************************

OLE1seq CCGCGGAGCCACCAGATGGTCAAGGCTGCCACCGCAGTCACCGCCGGCGGCTCCCTCCTC 120

OLE1trans CCGCGGAGCCACCAGATGGTCAAGGCTGCCACCGCAGTCACCGCCGGCGGCTCCCTCCTC 120

************************************************************

OLE1seq GTCCTCTCCGGCCTCACCCTCGCCGCCACCGTCATCGCCCTCACCATCGCCACCCCGCTC 180

OLE1trans GTCCTCTCCGGCCTCACCCTCGCCGCCACCGTCATCGCCCTCACCATCGCCACCCCGCTC 180

************************************************************

OLE1seq CTCGTCATCTTCAGCCCCGTGCTCGTGCCCGCGGCCCTGGCCGTGTTCGCCCTGGCTGGA 240

OLE1trans CTCGTCATCTTCAGCCCCGTGCTCGTGCCCGCGGCCCTGGCCGTGTTCGCCCTGGCTGGA 240

************************************************************

OLE1seq GGGTTCCTCGCGTCGGGCGGGTTCGGGGTGGCCGCCCTCAGCGTGCTGTCGTGGATCTAC 300

OLE1trans GGGTTCCTCGCGTCGGGCGGGTTCGGGGTGGCCGCCCTCAGCGTGCTGTCGTGGATCTAC 300

************************************************************

OLE1seq AAGTACATGACCGGGAAGCACCCGGTCGGGGCTGACCAGCTCGACACTGCGCGCACGAAG 360

OLE1trans AAGTACATGACCGGGAAGCACCCGGTCGGGGCTGACCAGCTCGACACTGCGCGCACGAAG 360

************************************************************

OLE1seq CTCGCGGGCAAGGCAAGGGACATGAAGGACAGGGTGGACCATAATGTCTCCGTTGCGCAA 420

OLE1trans CTCGCGGGCAAGGCAAGGGACATGAAGGACAGGGTGGACCATAATGTCTCCGTTGCGCAA 420

************************************************************

OLE1seq AGCTCT--- 426

OLE1trans AGCTCTTAG 429

******

1. **DGAT2:**

DGAT2seq ------------------------------------------------------------

DGAT2trans ATGTCGTCTGAATCCAACGGCGACGTCAGGCGGCGGAGATCGCCGTCGTCCGAAGCAGAG 60

DGAT2seq -----------------------AGAGTTCAAGGGCACTCGAGGATCGCTTATGAATTCC 37

DGAT2trans TCGGACGCGCCCCCGACCGCGGCAGAGTTCAAGGGCACTCGAGGATCGCTTATGAATTCC 120

*************************************

DGAT2seq ATTATTGCAATTGTTCTGTGGCTCGGGTCGGTCCATCTCATCGTCTCGATCGTCCTCGCC 97

DGAT2trans ATTATTGCAATTGTTCTGTGGCTCGGGTCGGTCCATCTCATCGTCTCGATCGTCCTCGCC 180

************************************************************

DGAT2seq TCGTTTTTCTTCCTCCCCTTTCCCAAATCACTCGGAGGAATTGTGTTGCTCTTCGTATTT 157

DGAT2trans TCGTTTTTCTTCCTCCCCTTTCCCAAATCACTCGGAGGAATTGTGTTGCTCTTCGTATTT 240

************************************************************

DGAT2seq ATGGTGATTCCGATTAATGAGCGGAGCAGATGGGGCCGGAATTTGGCCAGGTATATATGT 217

DGAT2trans ATGGTGATTCCGATTAATGAGCGGAGCAGATGGGGCCGGAATTTGGCCAGGTATATATGT 300

************************************************************

DGAT2seq TTCCGGTGGCGTTGCATGTGGAGAACATCAAAGCCTTTGATCCCAATGAAGCATATGTTT 277

DGAT2trans TTCCGGTGGCGTTGCATGTGGAGAACATCAAAGCCTTTGATCCCAATGAAGCATATGTTT 360

************************************************************

DGAT2seq TTGGTTATGAACCACATTCAGTTTGGCCTATTGGAGTAATTGCGATAGCAGATCTTACTG 337

DGAT2trans TTGGTTATGAACCACATTCAGTTTGGCCTATTGGAGTAATTGCGATAGCAGATCTTACTG 420

************************************************************

DGAT2seq GTTTCATGCCTCTTCCGAAGATCAAGGTTCTTGCAAGTTCTGCTGTGTTCTACACTCCCT 397

DGAT2trans GTTTCATGCCTCTTCCGAAGATCAAGGTTCTTGCAAGTTCTGCTGTGTTCTACACTCCCT 480

************************************************************

DGAT2seq TCATGCGACATTTATGGACGTGGTTGGGACTTTCAGCTGCTACAAGGAAAAATTTTACTG 457

DGAT2trans TCATGCGACATTTATGGACGTGGTTGGGACTTTCAGCTGCTACAAGGAAAAATTTTACTG 540

************************************************************

DGAT2seq CACTTTTGTCATCTGGTTATAGCTGCATTATAATTCCAGGAGGAGTCCAGGAGGCCTGCT 517

DGAT2trans CACTTTTGTCATCTGGTTATAGCTGCATTATAATTCCAGGAGGAGTCCAGGAGGCCTGCT 600

************************************************************

DGAT2seq ATATGGAGCATGGCTCTGAGGTTGCGTTTCTACAAAGCAGAAAAGGATTTGTACGAATTG 577

DGAT2trans ATATGGAGCATGGCTCTGAGGTTGCGTTTCTACAAAGCAGAAAAGGATTTGTACGAATTG 660

************************************************************

DGAT2seq CTATAGAGACTGGCAAACCTCTGGTGCCTGTTTTCTGCTTTGGACAGACTGATGTATACA 637

DGAT2trans CTATAGAGACTGGCAAACCTCTGGTGCCTGTTTTCTGCTTTGGACAGACTGATGTATACA 720

************************************************************

DGAT2seq AGTGGTGGAGACCAGGTGGGAAACTCTTTAGGGAGTTCTCCAGAGCCATAAAGTTCACAC 697

DGAT2trans AGTGGTGGAGACCAGGTGGGAAACTCTTTAGGGAGTTCTCCAGAGCCATAAAGTTCACAC 780

************************************************************

DGAT2seq CCATTGTGTTCTGGGGCGTACTAGGTTCACCTCTACCTTTTAGACAGCCGCTCCATGTGG 757

DGAT2trans CCATTGTGTTCTGGGGCGTACTAGGTTCACCTCTACCTTTTAGACAGCCGCTCCATGTGG 840

************************************************************

DGAT2seq TGGTTGGTGAACCAATTCTG---------------------------------------- 777

DGAT2trans TGGTTGGTGAACCAATTCTGGTGAAGAAAAATTCTCAACCTACTAAAGAAGAGGTTATGG 900

********************

DGAT2seq ------------------------------------------------------------

DGAT2trans AGGTCCATGCCCGGTTTGTGGAAGCGCTTCAAGATCTTTTCCAAAGACACAAAGCAAGGG 960

DGAT2seq -----------------------------------

DGAT2trans TCGGCCACCCAGACTTGCAGCTTAGGATTCTATAA 995

1. **DGAT3:**

DGAT3seq -----------------------------------------------CACCGCTGCTGCT 13

DGAT3trans ATGGACGCCGCTGCCATGGCTCTCCAGCAACCGATTCGCTTTCCAAACACCGCTGCTGCT 60

*************

DGAT3seq TCATCGTCGACGAAGGAACTCGGGAATTATGCTGGTAATCTCGTTCGATTGCCGCGGGGG 73

DGAT3trans TCATCGTCGACGAAGGAACTCGGGAATTATGCTGGTAATCTCGTTCGATTGCCGCGGGGG 120

************************************************************

DGAT3seq AGGACTAAAAACAGAGTATTGTCCTCTGGATTCTGCGATCGGGGCCATCTCCAATACTAT 133

DGAT3trans AGGACTAAAAACAGAGTATTGTCCTCTGGATTCTGCGATCGGGGCCATCTCCAATACTAT 180

************************************************************

DGAT3seq AGCTCTAGCTTCGATTCCGAGGAGGGGGTTTCAGGGAGGAGGATGAGTGTGATGAATGGG 193

DGAT3trans AGCTCTAGCTTCGATTCCGAGGAGGGGGTTTCAGGGAGGAGGATGAGTGTGATGAATGGG 240

************************************************************

DGAT3seq AAGGAAGTGAAGAGCGTTAAGGAGAAGTCGGTGAAGAAGATGAAGAAGAAGCAGTTGAAA 253

DGAT3trans AAGGAAGTGAAGAGCGTTAAGGAGAAGTCGGTGAAGAAGATGAAGAAGAAGCAGTTGAAA 300

************************************************************

DGAT3seq TTGCTTAAGGGATTGTCTCGGGATTTGTCAACCTTTTCTCAGATGGGATTTGGAATGGAT 313

DGAT3trans TTGCTTAAGGGATTGTCTCGGGATTTGTCAACCTTTTCTCAGATGGGATTTGGAATGGAT 360

************************************************************

DGAT3seq TCTGATAGCTCGCTCGTTGATCAGATTAAAGGAAACATGATCACGGAAGCAACACAGCTT 373

DGAT3trans TCTGATAGCTCGCTCGTTGATCAGATTAAAGGAAACATGATCACGGAAGCAACACAGCTT 420

************************************************************

DGAT3seq TTGCTGGAGCAGCTGCAGAAGGTGAAGGCAGAAGAGAAAGAAGCGAAAAAAAGAATCAAG 433

DGAT3trans TTGCTGGAGCAGCTGCAGAAGGTGAAGGCAGAAGAGAAAGAAGCGAAAAAAAGAATCAAG 480

************************************************************

DGAT3seq GAAGAGAAAGCAAGAATGAAGGCTGCAGCTAGAGCACAAATTGGGGCTAACTGCGAGATG 493

DGAT3trans GAAGAGAAAGCAAGAATGAAGGCTGCAGCTAGAGCACAAATTGGGGCTAACTGCGAGATG 540

************************************************************

DGAT3seq TCATCATCATCAAGTTCTTCCTCCGAATCAAGCGACAGTGAATGCGGAGAAGTCGTCGAC 553

DGAT3trans TCATCATCATCAAGTTCTTCCTCCGAATCAAGCGACAGTGAATGCGGAGAAGTCGTCGAC 600

************************************************************

DGAT3seq ATGAGTAGCCTCAAGCGCGCAACACCTACAAAAACCATCTTACAAGAAGCCAAGGTGGTG 613

DGAT3trans ATGAGTAGCCTCAAGCGCGCAACACCTACAAAAACCATCTTACAAGAAGCCAAGGTGGTG 660

************************************************************

DGAT3seq GTGGAGGAGGAGACAGCTCCATCATATCCAGCTCCGATCATACCAACCATTCCACCCTCG 673

DGAT3trans GTGGAGGAGGAGACAGCTCCATCATATCCAGCTCCGATCATACCAACCATTCCACCCTCG 720

************************************************************

DGAT3seq TTCGAAGCTGATGCTGCACCGGCCTCTCTTCTCCCCTCGCCCGAGGAGGAGCAGCCTAGC 733

DGAT3trans TTCGAAGCTGATGCTGCACCGGCCTCTCTTCTCCCCTCGCCCGAGGAGGAGCAGCCTAGC 780

************************************************************

DGAT3seq ACTTCAAGCAGAGTGCAGGATATAAGCTGCTCCGTTGCTCCATCTTGCTCGAAGAAGATT 793

DGAT3trans ACTTCAAGCAGAGTGCAGGATATAAGCTGCTCCGTTGCTCCATCTTGCTCGAAGAAGATT 840

************************************************************

DGAT3seq GAGGTGTGTATGGGAGGCAAGTGCAAGAAATCGGGCGCCGGTGCTCTGCTGGAGGAGTTC 853

DGAT3trans GAGGTGTGTATGGGAGGCAAGTGCAAGAAATCGGGCGCCGGTGCTCTGCTGGAGGAGTTC 900

************************************************************

DGAT3seq AGGAGAGCTGTGGGGATTGAAGGTGCAGTCTCAGGGTGCAAATGTATGGGGAAGTGCAGG 913

DGAT3trans AGGAGAGCTGTGGGGATTGAAGGTGCAGTCTCAGGGTGCAAATGTATGGGGAAGTGCAGG 960

************************************************************

DGAT3seq GATGGGCCTAATGTGAAGGTCGTGGGGCAGGAGTCGTCGAGCTCTTTGTGCATTGGTGTA 973

DGAT3trans GATGGGCCTAATGTGAAGGTCGTGGGGCAGGAGTCGTCGAGCTCTTTGTGCATTGGTGTA 1020

************************************************************

DGAT3seq GGGTTGGAGGATGTGAATGTGA-------------------------------------- 995

DGAT3trans GGGTTGGAGGATGTGAATGTGATCATGGCAAATTTCATTGGTGAACATCAGCAGATTGGT 1080

**********************

DGAT3seq ------------------

DGAT3trans TTTGCTGCTGCATCTTGA 1098

1. **Delta 15 Desaturase:**

Delta15des.seq ------------------------------------------------------------

Delta15des.trans ATGGCCGTCTCTTCCGGTGCCGACGCTGAGCACCACGGCCACGCCCAATACGAGCACCTC 60

Delta15des.seq ------------GCCGACAAATTCGACCCGGCCGCGCCTCCTCCGTTCAAGATCGCCGAC 48

Delta15des.trans GGCAAGCGCGCCGCCGACAAATTCGACCCGGCCGCGCCTCCTCCGTTCAAGATCGCCGAC 120

************************************************

Delta15des.seq ATCCGCGCCGCCATCCCGCCGCATTGCTGGGTCAAGGACCCCCTCCGCTCCCTCAGCTAC 108

Delta15des.trans ATCCGCGCCGCCATCCCGCCGCATTGCTGGGTCAAGGACCCCCTCCGCTCCCTCAGCTAC 180

************************************************************

Delta15des.seq GTCGCCTGGGATGTGTTCGTCGTCGCCGCGCTCCTCGCCGCCGCCGCCTTTTTCGACAGC 168

Delta15des.trans GTCGCCTGGGATGTGTTCGTCGTCGCCGCGCTCCTCGCCGCCGCCGCCTTTTTCGACAGC 240

************************************************************

Delta15des.seq TGGATCTTCTGGCCCATCTACTGGGCCGCCCAGGGCACCATGTTTTGGGCCTTGTTCGTC 228

Delta15des.trans TGGATCTTCTGGCCCATCTACTGGGCCGCCCAGGGCACCATGTTTTGGGCCTTGTTCGTC 300

************************************************************

Delta15des.seq CTCGGCCACGATTGTGGGCACGGGAGTTTTTCGGACAATACCACGCTGAATAACGTGGTG 288

Delta15des.trans CTCGGCCACGATTGTGGGCACGGGAGTTTTTCGGACAATACCACGCTGAATAACGTGGTG 360

************************************************************

Delta15des.seq GGACATGTACTACATTCCTCAATTCTTGTACCTTATCATGGATGGCGAATTAGCCATCGA 348

Delta15des.trans GGACATGTACTACATTCCTCAATTCTTGTACCTTATCATGGATGGCGAATTAGCCATCGA 420

************************************************************

Delta15des.seq ACACACCACCAGAATCATGGTCATGTGGAGAACGACGAGTCATGGGTTCCGCTGACTGAG 408

Delta15des.trans ACACACCACCAGAATCATGGTCATGTGGAGAACGACGAGTCATGGGTTCCGCTGACTGAG 480

************************************************************

Delta15des.seq AATTTATACAAGCAGCTGGATTTCTCCACCAAATTCTTGAGATACAAAATCCCATTCCCC 468

Delta15des.trans AATTTATACAAGCAGCTGGATTTCTCCACCAAATTCTTGAGATACAAAATCCCATTCCCC 540

************************************************************

Delta15des.seq ATGTTTGCCTACCCCCTATACTTGTGGTATAGAAGCCCCGGAAAAAGTGGATCTCACTTC 528

Delta15des.trans ATGTTTGCCTACCCCCTATACTTGTGGTATAGAAGCCCCGGAAAAAGTGGATCTCACTTC 600

************************************************************

Delta15des.seq AACCCATATAGTAGTTTGTTCAAACCCAATGAGAGAGATTTGGTGATCACTTCCACCATA 588

Delta15des.trans AACCCATATAGTAGTTTGTTCAAACCCAATGAGAGAGATTTGGTGATCACTTCCACCATA 660

************************************************************

Delta15des.seq TGTTGGGCTGCAATGGTTGCTTGTCTCCTCTATGCTTCCACCATTGTTGGCCCAACCATG 648

Delta15des.trans TGTTGGGCTGCAATGGTTGCTTGTCTCCTCTATGCTTCCACCATTGTTGGCCCAACCATG 720

************************************************************

Delta15des.seq TTGTTCAAGCTCTACGGCGTTCCTTATTTGATATTCGTTGTGTGGTTGGACACGGTTACA 708

Delta15des.trans TTGTTCAAGCTCTACGGCGTTCCTTATTTGATATTCGTTGTGTGGTTGGACACGGTTACA 780

************************************************************

Delta15des.seq TATCTGCACCACCATGGTTACGACAAGAAACTCCCTTGGTACCGCAGCAAGGAATGGAGT 768

Delta15des.trans TATCTGCACCACCATGGTTACGACAAGAAACTCCCTTGGTACCGCAGCAAGGAATGGAGT 840

************************************************************

Delta15des.seq TATTTACGTGGAGGATTGACGACAGTAGATCAAGACTATGGAATATTCAACAAAATTCAC 828

Delta15des.trans TATTTACGTGGAGGATTGACGACAGTAGATCAAGACTATGGAATATTCAACAAAATTCAC 900

************************************************************

Delta15des.seq CACGATATTGGCACCCATGTTGTTCACCACCTATTCCCTCAGATCCCACATTACCATTTA 888

Delta15des.trans CACGATATTGGCACCCATGTTGTTCACCACCTATTCCCTCAGATCCCACATTACCATTTA 960

************************************************************

Delta15des.seq GTGGAGGCGACGAGGGAGGCGAAAAGGGTGCTCGGAAATTACTACAGAGAGCCCAGAAAA 948

Delta15des.trans GTGGAGGCGACGAGGGAGGCGAAAAGGGTGCTCGGAAATTACTACAGAGAGCCCAGAAAA 1020

************************************************************

Delta15des.seq TCTGGAGCCGTT------------------------------------------------ 960

Delta15des.trans TCTGGAGCCGTTCCGTTTCACTTGGTTCCGACGTTGTTGAAAAGTCTAAGTAGAGATCAT 1080

************

Delta15des.seq ------------------------------------------------------------

Delta15des.trans TATGTGAGTGACAATGGAGACATAGTTTACTATCAAACAGATGGAGAACTATTTTCTTCT 1140

1. **OMEGA 3 DESATURASE:**

Omega3des.seq GCCGCCGCCGCCTATTTCAACAGTTGGATTGTTTGGCCATTGTACTGGTTTGCTCAGAGC 60

Omega3des.trans ------GCCGCCTATTTCAACAGTTGGATTGTTTGGCCATTGTACTGGTTTGCTCAGAGC 54

******************************************************

Omega3des.seq ACCATGTTCTGGGCTCTCTTTGTTCTTGGTCATGACTGTGGCCATGGAAGCTTTTCTAAC 120

Omega3des.trans ACCATGTTCTGGGCTCTCTTTGTTCTTGGTCATGACTGTGGCCATGGAAGCTTTTCTAAC 114

************************************************************

Omega3des.seq AATCCCAAGTTGAATAGTGTGTTTGGCCATTTCCTTCACTCTTCAATTTTGGTGCCCTAC 180

Omega3des.trans AATCCCAAGTTGAATAGTGTGTTTGGCCATTTCCTTCACTCTTCAATTTTGGTGCCCTAC 174

************************************************************

Omega3des.seq CATGGATGGAGAATTAGTCACAGAACTCATCATCAGAACCATGGGCATGTTGAGAATGAT 240

Omega3des.trans CATGGATGGAGAATTAGTCACAGAACTCATCATCAGAACCATGGGCATGTTGAGAATGAT 234

************************************************************

Omega3des.seq GAATCTTGG 249

Omega3des.trans ---------
