# Supplementary material for: Exploring Triacylglycerol Biosynthetic Pathway in Developing Seeds of Chia (Salvia hispanica L.): A Transcriptomic Approach
Source: PLoS One. 2015 Apr 13;10(4):e0123580. doi: 10.1371/journal.pone.0123580 (PMC4395390; doi:10.1371/journal.pone.0123580)
Supplement: S2 File — (DOCX) [file pone.0123580.s003.docx]

**Protein sequence alignment of some Chia lipid genes (Sh) with known oil seed plants showing maximum homology. [*Arabidopsis thaliana* (At), *Arachis hypogea* (Ah), *Perilla frutescens* (Pf), *Crepis alpine* (Ca), *Glycine max* (Gm) and *Brassica napus* (Bn)].**

1. **MGAT**

AhMgat ------------MGTSGGTPNFWGHMPEEEYYTSQGVRNTKSFFDTPHGKIFTQSFLPLD 48

At1G52760 MPSEAESSANSAPATPPPPPNFWGTMPEEEYYTSQGVRNSKSYFETPNGKLFTQSFLPLD 60

ShMgat -------------MSPENPSNFWGDTPEEEYYASQGVRNSKSYFDSPHGRLFTQSFLPLD 47

:. ..**** ******:******:**:*::*:*::*********

AhMgat LQPNEVKATVFMTHGYGSDTGWLFQKICINFATWGYAVFAADLLGHGRSDGLRCYLGDMD 108

At1G52760 G---EIKGTVYMSHGYGSDTSWMFQKICMSFSSWGYAVFAADLLGHGRSDGIRCYMGDME 117

ShMgat P-TRPVKASVFMTHGYGSDSSWMFQKFCISYAAWGYAVFAADMLGHGRSDGIRCYMGDLP 106

:*.:*:*:******:.*:***:*:.:::*********:********:***:**:

AhMgat KVASASLSFFLHVRRSEPYKDLPAFLFGESMGGLATLLMYFKSEPDTWTGLMFSAPLFVI 168

At1G52760 KVAATSLAFFKHVRCSDPYKDLPAFLFGESMGGLVTLLMYFQSEPETWTGLMFSAPLFVI 177

ShMgat KVAAASLAFFRSVRVSDEYKDLPAFLVGESMGGLATLLMYFQSEKDLWTGLIFSAPLFVI 166

***::**:** ** *: ********.*******.******:** : ****:********

AhMgat PEDMKPSRLHLFAYGLLFGWADTWAAMPDNKMVGKAIRDPEKLKIIASNPRRYTGPPRVG 228

At1G52760 PEDMKPSKAHLFAYGLLFGLADTWAAMPDNKMVGKAIKDPEKLKIIASNPQRYTGKPRVG 237

ShMgat PESMMPSKVHLFAYGMLFGLADTWAAMPDNKMVGKAIKDPEKLKVIASNPMRYTGKPRVG 226

**.* **: ******:*** *****************:******:***** **** ****

AhMgat TMRELLRVTQYVQDNFSKVTAPFLTVHGTSDGVTCPSSSKLLYEKASSEDKSLKLYEGMY 288

At1G52760 TMRELLRKTQYVQENFGKVTIPVFTAHGTADGVTCPTSSKLLYEKASSADKTLKIYEGMY 297

ShMgat TMRELLRQTEYAQNNFDKVTIPFFTAHGTSDGLAEWSGSQMLYDKASSEDKTLKLYEGMY 286

******* *:*.*:**.*** *.:*.***:**:: :.*::**:**** **:**:*****

AhMgat HSLIQGEPDESANLVLSDMREWIDQRVHRYGNK-- 321

At1G52760 HSLIQGEPDENAEIVLKDMREWIDEKVKKYGSKTA 332

ShMgat HSLIQGEPDENANLVLADMRAWIDERVERYGKKN- 320

**********.*::** *** ***::*.:**.*

1. **OLE1:**

AtOleosin MADTARGTHHDIIGR-DQYPMMGRDRDQYQMSGRGSDYSKSRQIAKAATAVTAGGSLLVL 59

ShOle1 MADQHYG----------QFQSRPHHLQQHHP--------RSHQMVKAATAVTAGGSLLVL 42

AhOleosin MSDQTRTGYGGGGSYGSSYGGGGTYGSSYGTSYDPSTNQPIRQAIKFMTASTIGVSFLIL 60

*:* .: ..: :* * ** * * *:*:*

AtOleosin SSLTLVGTVIALTVATPLLVIFSPILVPALITVALLITGFLSSGGFGIAAITVFSWIYKY 119

ShOle1 SGLTLAATVIALTIATPLLVIFSPVLVPAALAVFALAGGFLASGGFGVAALSVLSWIYKY 102

AhOleosin SGLILTGTVIGLIIATPLLVIFSPILVPAAITLALAAGGFLFSGGCGVAAIAALSWLYSY 120

*.* *..***.* :**********:**** ::: *** *** *:**::.:**:*.*

AtOleosin ATGEHPQGSDKLDSARMKLGSKAQDLKDRAQYYGQQHTGGEHDRDRTRGGQHTT--- 173

ShOle1 MTGKHPVGADQLDTARTKLAGKARDMKDRVDHN-------------VSVAQSS---- 142

AhOleosin VTGKHPAGSDRLDYAKGVIADKARDVKDRAKDYAG-----------AGRAQEGTPGY 166

**:** *:*:** *: :..**:*:***.. . .*

1. **DGAT2:**

AhDgat2 --MEDRGNVT-VAPPAEEKVFRSTEVFAPESSSKSKGFKTTLALALWLGAIHFNGALMLF 57

AtDgat2 --------------------MGGSREFRAEEHSN--QFHSIIAMAIWLGAIHFNVALVLC 38

ShDgat2 MSSESNGDVRRRRSPSSEAESDAPPTAAEFKGTRGSLMNSIIAIVLWLGSVHLIVSIVLA 60

.. . :. ::: :*:.:***::*: :::*

AhDgat2 ALLFLPLSKALLVFALLFVFMVIPIDEKSKFGRKLSRYICKNACSYFPITLHVEDIKAFN 117

AtDgat2 SLIFLPPSLSLMVLGLLSLFIFIPIDHRSKYGRKLARYICKHACNYFPVSLYVEDYEAFQ 98

ShDgat2 SFFFLPFPKSLGGIVLLFVFMVIPINERSRWGRNLARYICKHAVGYFPVALHVENIKAFD 120

:::*** . :* : ** :*:.***:.:*::**:*:*****:* .***::*:**: :**:

AhDgat2 SNRAYVFGFEPHSVLPIGVVALADNTGFMPLPKIKVLASSAVFYTPFLRHIWTWLGLTPA 177

AtDgat2 PNRAYVFGYEPHSVLPIGVVALCDLTGFMPIPNIKVLASSAIFYTPFLRHIWTWLGLTAA 158

ShDgat2 PNEAYVFGYEPHSVWPIGVIAIADLTGFMPLPKIKVLASSAVFYTPFMRHLWTWLGLSAA 180

.*.*****:***** ****:*:.* *****:*:********:*****:**:******:.*

AhDgat2 TKKNFLSLLDNGYSCILIPGGVQETFLMEHGTETAYLKARKGFIRIAMQKGQPLVPVFCF 237

AtDgat2 SRKNFTSLLDSGYSCVLVPGGVQETFHMQHDAENVFLSRRRGFVRIAMEQGSPLVPVFCF 218

ShDgat2 TRKNFTALLSSGYSCIIIPGGVQEACYMEHGSEVAFLQSRKGFVRIAIETGKPLVPVFCF 240

::*** :**..****:::******: *:*.:* .:*. *:**:***:: *.********

AhDgat2 GQSDIYKWWKPGGKLILNFARAIKFTPIYFWGIFGSPIPFKHPMYVVVGRPIELDKNPEP 297

AtDgat2 GQARVYKWWKPDCDLYLKLSRAIRFTPICFWGVFGSPLPCRQPMHVVVGKPIEVTKTLKP 278

ShDgat2 GQTDVYKWWRPGGKLFREFSRAIKFTPIVFWGVLGSPLPFRQPLHVVVGEPILVKKNSQP 300

**: :****:*. .* :::***:**** ***::***:* ::*::****.** : *. :*

AhDgat2 TTEEVATVHSQFVASLQDLFERYKARAGYPNLELRIV 334

AtDgat2 TDEEIAKFHGQYVEALRDLFERHKSRVGY-DLELKIL 314

ShDgat2 TKEEVMEVHARFVEALQDLFQRHKARVGHPDLQLRIL 337

* **: .*.::* :*:***:*:*:*.*: :*:*:*:

1. **DGAT3:**

AtDgat MEVSGVVLRQIPCVSSGSVAGLRLVSEFSGNTRTVGFRTRRFRGIVCNNEFADKGHVNYY 60

AhDgat MEVSGTVLRNITCPSFSMHVSSRGG---GGGCVSVPVRLRKKAVVRCCCGFSDSGHVRYY 57

ShDgat3 MDAAAMALQQPIRFPNTAAASSSTKELGNYAGNLVRLPRGRTKNRVLSSGFCDRGHLQYY 60

*:.:. .*:: . .. . * . : *.* **:.**

AtDgat IEPTRC------------GEEKEKVKVMEKEKKALKKKAKVLKSLSKNLDMFSSIGFGLD 108

AhDgat ------------------GDEKKKKEENGTAVLSTKKKLKMLK----------------- 82

ShDgat3 SSSFDSEEGVSGRRMSVMNGKEVKSVKEKSVKKMKKKQLKLLKGLSRDLSTFSQMGFGMD 120

. :: * . **: *:**

AtDgat PEAGLVGEIQTKTISEATEILVKQLEQLKAEEKILKKQRKEEK-AKAKAMKKMTEMDSES 167

AhDgat -KRVLFDDLQGNLTSDAAEVLMKQLEQARAEEKELKRKRKQEKEAKLKASKMNTNPDCES 141

ShDgat3 SDSSLVDQIKGNMITEATQLLLEQLQKVKAEEKEAKKRIKEEKARMKAAARAQIGANCEM 180

. *..::: : ::*:::*::**:: :**** *:: *:** * : :.*

AtDgat SSSS---ESSDSDCDKGKVVDMSS-LRNKAKPVLEPLQPEATVATLPRIQEDAISCKNTS 223

AhDgat SSSSSESESSESECDN-EVVDMKKNMKVGVAVADSPPKAETMIYTPPLLPEDVSVNDHHH 200

ShDgat3 SSSSS-SSSESSDSECGEVVDMSS-LKRATPTKTILQEAKVVVEEETAPSYPAPIIPTIP 238

**** .*..*:.: :****.. :: . :.:. : . .

AtDgat EALQIALQTSTIFPS-MANPGQTLKTVEAVSVVGLP--LNRVEVCMGGKCKRSGGALLLD 280

AhDgat KIMELFSRNNDISVG-SINGSLKNENTAVITTESIP--QKRIEVCMGNKCKKSGSIALLQ 257

ShDgat3 PSFEADAAPASLLPSPEEEQPSTSSRVQDISCSVAPSCSKKIEVCMGGKCKKSGAGALLE 298

:: : . : . . . :: * :::*****.***:**. **:

AtDgat EFQRAMTGFEG-----SAVACKCMGKCRDGPNVRVVKETDAVMTD----SVRTPSKTLCV 331

AhDgat EFER-VVGAEGGADAAAVVGCKCMGKCKSAPNVRIQNSTADKIAEGFNDSVKVPANPLFV 316

ShDgat3 EFRR-AVGIEG-----AVSGCKCMGKCRDGPNVKVVGQ--------------ESSSSLCI 338

**.* .* ** :. .*******:..***:: . .:..* :

AtDgat GVGLQDVETIVTSFFDEECSREGLGSVSY 360

AhDgat GVALEDVETIVARFLGEN--QESTNE--- 340

ShDgat3 GVGLEDVNVIMANFIGEHQQIGFAAAS-- 365

**.*:**:.*:: *:.*.

1. **DELTA 15 DESATURASE:**

PfOmega-3 MAVSSGARLSKSGADGEVFDGQQQYEGIGKRAADKFDPAAPPPFKIADIRAAIPAHCWVK 60

ShDelta-15 MAVSS-------GADAEHH-GHAQYEHLGKRAADKFDPAAPPPFKIADIRAAIPPHCWVK 52

CaOmega-3 MAVSS------PDATEKLNQASPLVNGVQK-NQEEFDPSAPPPFKVADIRAAIPSHCWVK 53

***** .* : . : : * ::***:******:********.*****

PfOmega-3 NPWRSLSYVVWDVAAVFALLAAAVYIN-SWAFWPVYWIAQGTMFWALFVLGHDCGHGSFS 119

ShDelta-15 DPLRSLSYVAWDVFVVAALLAAAAFFD-SWIFWPIYWAAQGTMFWALFVLGHDCGHGSFS 111

CaOmega-3 NPWRSLSYVLRDILVISSLVAIAVIFKTSSWVWPIYWIAQGTMFWAIFVLGHDCGHGSFS 113

:* ****** *: .: :*:* *. :. * .**:** ********:*************

PfOmega-3 DNTTLNNVVGHVLHSSILVPYHGWRISHRTHHQNHGHVEKDESWVPLPENLYKKLDFSTK 179

ShDelta-15 DNTTLNNVVGHVLHSSILVPYHGWRISHRTHHQNHGHVENDESWVPLTENLYKQLDFSTK 171

CaOmega-3 DNPNLNSVVGHILHSSILVPYHGWRISHRTHHQNHGHVENDESWVPLTEKTYNTLDASTK 173

**..**.****:***************************:*******.*: *: ** ***

PfOmega-3 FLRYKIPFPMFAYPLYLWYRSPGKTGSHFNPYSDLFKPNERGLIVTSTMCWAAMGVFLLY 239

ShDelta-15 FLRYKIPFPMFAYPLYLWYRSPGKSGSHFNPYSSLFKPNERDLVITSTICWAAMVACLLY 231

CaOmega-3 LLRFKVPFPLFAYPLYLWYRSPGKSGSHFNPYSDMFSPNERSYIVTSTLCWSLMVANLVF 233

:**:*:***:**************:********.:*.****. ::***:**: * . *::

PfOmega-3 ASTIVGPNMMFKLYGVPYLIFVMWLDTVTYLHHHGYDKKLPWYRSKEWSYLRGGLTTVDQ 299

ShDelta-15 ASTIVGPTMLFKLYGVPYLIFVVWLDTVTYLHHHGYDKKLPWYRSKEWSYLRGGLTTVDQ 291

CaOmega-3 LSIVIGPTLLFNLYGIPYLIFVMWLDFVTYLHHHGHEQKLPWYRGKEWSYLRGGLTTVDR 293

* ::**.::*:***:******:*** ********:::******.**************:

PfOmega-3 DYGFFNKIHHDIGTHVIHHLFPQIPHYHLVEATREAKRVLGNYYREPRKSGPVPLHLIPA 359

ShDelta-15 DYGIFNKIHHDIGTHVVHHLFPQIPHYHLVEATREAKRVLGNYYREPRKSGAVPFHLVPT 351

CaOmega-3 DYGIFNNIHHDIGTHVIHHLFPQIPHYHLIEATMAAKSVLGNYYREPKKSGWIPVHLMDN 353

***:**:*********:************:*** ** *********:*** :*.**:

PfOmega-3 LLKSLGRDHYVSDNGDIVYYQTDDELFPSKKI- 391

ShDelta-15 LLKSLSRDHYVSDNGDIVYYQTDGELFSSKEI- 383

CaOmega-3 LVKSVKQDHYVSDAGEVVYYQTDYRMIG-KKIE 385

*:**: :****** *::****** .:: *:*

1. **OMEGA 3 DESATURASE:**

GmOmega3des MATWVLSECGLRPLPPVFPRSTRPISCQKPSKSRFLSTNKGVPDLNLQARGLTCCSFRDR 60

ShOmega3des ------------------------------------------------------------

BnOmega3des ------------------------------------------------------------

GmOmega3des KWELGVSAPLKFATNEGEEEERTNGANNGVGEEVSEFDPSAPPPFKLADIRAAIPKHCWV 120

ShOmega3des ------------------------------------------------------------

BnOmega3des ------------------------------------------------------------

GmOmega3des KDPWKSMSYVVRDVIVVFGLAAAAAYLNNWVVWPLYWAAQGTMFWALFVLGHDCGHGSFS 180

ShOmega3des ------------------------------------------MFWALFVLGHDCGHGSFS 18

BnOmega3des ------MSYVVRELAIVFALAAGAAYLNNWLVWPLYWIAQGTMFWALFVLGHDCGHGSFS 54

******************

GmOmega3des NNPKLNSVAGHLLHSSILVPYHGWRISHRTHHQNHGHVENDESWHPLPEKIFKSLDNVTR 240

ShOmega3des NNPKLNSVFGHFLHSSILVPYHGWRISHRTHHQNHGHVENDESW---------------- 62

BnOmega3des NDPRLNSVVGHLLHSSILVPYHGWRISHRTHHQNHGHVENDESWHPMSEKIYKSLDKPTR 114

*:*:**** **:********************************

GmOmega3des ILRFTLPFPLLAYPIYLWSRSPGKTGSHFNPDSDLFVPSERKDVITSTICWTAMAALLVG 300

ShOmega3des ------------------------------------------------------------

BnOmega3des FFRFTLPLVMLAYPFYLWARSPGKKGSHYHPDSDLFLPKERNDVLTSTACWTAMAVLLVC 174

GmOmega3des LGFVMGPVQLLKLYGIPYAIFVMWLDLVTYLHHHGHEDKLPWYRGEEWSYLRGGLTTIDR 360

ShOmega3des ------------------------------------------------------------

BnOmega3des LNFVMGPMQMLKLYVIPYWINVMWLDFVTYLHHHGHEDKLPWYRGKEWSYLRGGLTTLDR 234

GmOmega3des DYGWINNIHHDIGTHVIHHLFPQIPHYHLIEATEAAKPVLGQYYREPKKSSPLPIYLIGE 420

ShOmega3des ------------------------------------------------------------

BnOmega3des DYGLINNIHHDIGTHVIHHLFPQIPHYHLVEATEAAKPVLGKYYREPDKSGPLPLHLLGI 294

GmOmega3des LLRSMKKDHFVSDSGDIVYYQTDPTLS--SSSTSQ 453

ShOmega3des -----------------------------------

BnOmega3des LAKSIKEDHFVSDEGDVVYYEADPNLYGEIKVTAE 329
